# PET Radiopharmaceuticals for Imaging Chemotherapy-Induced Cardiotoxicity

**DOI:** 10.1007/s11886-020-01315-z

**Published:** 2020-06-19

**Authors:** Jothilingam Sivapackiam, Monica Sharma, Thomas H. Schindler, Vijay Sharma

**Affiliations:** 1 Mallinckrodt Institute of Radiology, Washington University School of Medicine, PO Box 8225, 510 S. Kingshighway Blvd, St. Louis, MO 63110 USA; 2 Departments of Medicine, Cardiology and Nuclear Medicine, Washington University School of Medicine, St. Louis, MO 63110 USA; 3 Department of Neurology, Washington University School of Medicine, St. Louis, MO 63110 USA; 4 Department of Biomedical Engineering, School of Engineering & Applied Science, Washington University, St. Louis, MO 63105 USA

**Keywords:** Cardiotoxicity, PET radiopharmaceuticals, Anthracycline, ABCB1, ABCG2, Galmydar

## Abstract

**Purpose of Review:**

Currently, cardiotoxicity is monitored through echocardiography or multigated acquisition scanning and is defined as 10% or higher LVEF reduction. The latter stage may represent irreversible myocardium injury and limits modification of therapeutic paradigms at earliest stages. To stratify patients for anthracycline-related heart failure, highly sensitive and molecularly specific probes capable of interrogating cardiac damage at the subcellular levels have been sought.

**Recent Findings:**

PET tracers may provide noninvasive assessment of earliest changes within myocardium. These tracers are at nascent stages of development and belong primarily to (a) mitochondrial potential-targeted and (b) general ROS (reactive oxygen species)-targeted radiotracers. Given that electrochemical gradient changes at the mitochondrial membrane represent an upstream, and earliest event before triggering the production of the ROS and caspase activity in a biochemical cascade, the former category might offer interrogation of cardiotoxicity at earliest stages exemplified by PET imaging, using ^18^F-Mitophos and ^68^Ga-Galmydar in rodent models.

**Summary:**

Both categories of radiotracers may provide tools for monitoring chemotherapy-induced cardiotoxicity and interrogating therapeutic efficacy of cardio-protectants.

## Introduction

Over the last decade, successes in chemotherapy have contributed enormously to improvements in survival rates of cancer patients. During this intermediate period, the design and development of targeted therapeutics and characterization of novel biomarkers that mediate the pathophysiology of tumor biology in general, and their distant metastases in particular, have both demonstrated unparalleled growth, thereby advancing substantially the fields of medicinal chemistry, chemical, and tumor biology. Among these discoveries, while therapeutics directed at signaling pathways (angiogenesis) to impede proliferation of tumor cells via kinase inhibitors have shown promising outcomes, the discovery of immune check-point inhibitors (ICIs) have also offered remarkable tools in kits of molecular oncologists to treat both solid- and hematological tumors. Among these ICIs, agents targeting cytotoxic T lymphocyte-associated antigen 4 (CTLA-4), programmed cell death receptor-1 (PD-1), and programmed cell death ligand-1 (PD-L1) have found remarkable clinical success [1, 2]. The mechanism of action for these agents involve inhibiting the tumor cells from inactivating the immune system, thereby restoring the immune system role against the infiltrating tumor cells, thus impacting positively the healthcare landscape by enhancing survival rates in patient populations with typically poor outcomes using other treatment paradigms [3, 4]. However, each therapeutic advance has encountered its own challenges; therefore, ICIs also could not escape that fate. Importantly, the stimulation of the immune system is not without risk and is known to be associated with multi-organ adverse events [5]. These adverse events have been shown to occur with a variable frequency depending on the type of ICI, the type and location of the tumor, and host-traits [6]. Despite these advances, anthracyclines (exemplified by Doxorubicin or related analogues) either as primary intervention or as a combination therapy continue to be the main workhorse of patient management plans in molecular oncology clinics due to their utility in numerous malignancies (but not limited to), such as acute leukemia, non-Hodgkin lymphoma, breast cancer, and sarcomas. However, the benefits of these advances in cancer chemotherapy have been offset by concerns about cardiotoxicity-related adverse effects induced by administered anthracyclines or combination therapy. Most chemotherapeutic agents damaging myocardium often affect the circulatory vessels, impair coronary endothelial function, and can potentially induce left ventricular dysfunction or heart failure by generation of reactive oxygen species, and apoptosis [7]. Cardiotoxicity may be acute, which occurs either during or immediately following treatment and can be either transient or chronic, and has been typically categorized into type I (early onset) and type II (late onset) [8]. While the type I cardiotoxicity has been considered to be irreversible cardiac cell injury, and normally caused by anthracyclines, and analogues thereof including combination therapy, the type II cardiotoxicity is typically induced by molecularly targeted antibodies [9•].

Cardio-oncology is a rapidly emerging field for managing cancer-therapy-related cardiotoxicity, the consensus around using left ventricle ejection fraction (LVEF) is one of the metrics, and is evolving criterion. For example, the Cardiac Review and Evaluation Committee of trastuzumab-associated cardiotoxicity defines it by symptoms of heart failure, decline of LVEF, either as symptomatic LVEF decrease from ≥ 5 to < 55% or an asymptomatic reduction of LVEF ≥ 10 to < 55% [10]. The American Society of Echocardiography and European Association of Cardiovascular Imaging define cardiotoxicity as global longitudinal strain (GLS) with a 10–15% early reduction. Finally, FDA defines LVEF drop < 40–45% or is 40–49% with a ≥ 10% absolute decrease below baseline with anti-HER2 targeted therapy as a benchmark for monitoring cardiotoxicity [11•]. While the consensus among clinical community would continue to emerge on defining features of cardiotoxicity, molecular imaging agents capable of offering early detection of cardiotoxicity in vivo could offer powerful noninvasive tools for understanding role of different biomarkers regulating its pathophysiology, while simultaneously allowing opportunities for stratification of therapeutic choices for guiding patient management in the twenty-first century.

## Mechanisms and Biomarkers

While mechanisms of action for anthracyclines are multifactorial, the consensus among scientific community is that chemotherapy can potentially trigger increased production of reactive oxygen and reactive nitrogen species, lipid peroxidation, inflammation, induced cardiomyocyte apoptosis, interstitial fibrosis, and abnormal signaling of epidermal growth factor as well as β-arrestin (the highly conserved family of cytosolic adaptor proteins that contribute to many immune functions by orchestrating the desensitization and internalization of cell-surface G protein-coupled receptors (GPCRs) via well-studied canonical interactions), inhibition of nuclear topoisomerase II β, induced DNA damage, inhibition of vascular endothelial growth factor signaling, defective mitochondrial biogenesis, and calcium overloading [12–14]. Among the intracellular organelles, while maintaining their role as energy powerhouse, mitochondria have an essential role in myocardial tissue homeostasis; thus, either deterioration or partial impairment in their normal function leads to cardiomyocyte and endothelial cell death, consequently thus inducing cardiovascular dysfunction [15]. For example, the antiretroviral nucleoside reverse transcriptase inhibitors such as zidovudine may cause cardiac mitochondrial dysfunction through inhibition of DNA polymerase-gamma and induction of mitochondrial DNA mutations, thus leading to cardiomyopathy [16]. Overall, cardiac adverse effects can primarily be classified into 2 categories: (a) functional and (b) structural effects. Of note, seriously altered function may be completely dissociated from the structural effect, especially at an early stage [17]. Other than the functional deterioration, anthracyclines have also been shown to damage several proteins regulating cardiac muscle contractility including titin, the myofilament forming protein that regulates cardiac function leading to systolic and diastolic dysfunction [18]. Finally, the inter-individual variability in the susceptibility to chronic anthracycline-induced cardiotoxicity has also been reported. Therefore, the genetic variants with occurrence of drug-induced cardiotoxicity have also been identified, thus suggesting a potential role for gene polymorphisms that may control the metabolism of anthracyclines [19], detoxification of free radicals, and alterations in physiological iron levels. Finally, SNPs in Her2/neu Pro 1170 Ala polymorphism have also been identified in a subset of patients with increased risk of cardiotoxicity from trastuzumab therapy and postulated to be deployed with other risk biomarkers for stratification of patients [20]. These factors suggest challenges in encountering cardiotoxicity, while presenting opportunities to design new probes potentially capable of monitoring noninvasively cardiac dysfunction to manage cancer treatment paradigms in twenty-first century.

Currently, commonly used noninvasive diagnostic biomarkers to assess anthracycline-induced cardiotoxicity are cardiac troponins, brain natriuretic peptide, and N-terminal fragment of natriuretic peptide. Of note, persistent elevation of cardiac troponin I levels post 1-month treatment of anthracycline has also led to prevalence of more cardiac adverse events in 84% patients at 3 years compared with patients showing normal levels of cardiac troponin I levels. Additionally, circulating microRNAs also offer promising noninvasive tools and have been evaluated in children and young adults treated with anthracycline chemotherapy [21•]. Finally, other less commonly used biomarkers for cardiotoxicity include cytostatin C, galectin-3, interleukin 6, tumor necrosis factor α (TNFα), myeloperoxidase, and C-reactive protein [22]. However, due to their potential for being influenced by numerous micro-environmental factors and non-cardiovascular diseases, these circulating biomarkers lack desired sensitivity and disease specificity.

To interrogate cardiotoxicity at a molecular level, molecular imaging enables visualization, characterization, and quantification of biomarkers or physiological processes at cellular and subcellular levels in vivo. Therefore, it is conceivable that cardiac nuclear imaging agents (PET and SPECT) may provide versatile diagnostic tools to detect cardiotoxicity at earlier stages, while enabling interrogation of therapeutic efficacy to afford stratification of chemotherapeutic choices in molecular oncology for better management of cardiotoxicity concerns.

## Nuclear Imaging Tracers

Currently, cardiotoxicity is evaluated by echocardiography or multigated acquisition scanning and is typically defined as 10% or higher reduction of LVEF. However, a loss of such a magnitude in contraction function can be an indication of significant irreversible myocardium injury, thus substantially diminishing opportunities for interventions or modification of treatment plans. Therefore, interrogation of myocardium abnormalities at subcellular level may provide early and sensitive readout of drug-induced cardiotoxicity. Although pathophysiology of chemotherapy induced heart failure is a complex phenomenon involving multiple intersecting biochemical pathways, anthracycline-induced effects at a subcellular level have been known to be attributed to mitochondrial dysfunction or partial impairment in its normal function and elevated levels of oxidative stress. Arguably, nuclear imaging tracers have been under development based upon both themes. While the first category of tracers find their roots in application of fluorescent lipophilic cations and their utility in reporting about alterations in mitochondrial potential in cells [23], the second category of tracers has focused upon exploiting utility of dihydroethidium derivatives capable of detecting ROS in vivo. While the fluorescent lipophilic cations such as tetramethylrhodamine ethyl ester (TMRE) and Rhodamine 123 lack the desired depth penetration for whole body scanning, ^99m^Tc-Sestamibi, a hydrophobic and monocationic technetium(I) octahedral complex, has been routinely used in clinic as a myocardial perfusion imaging agent. Importantly, ^99m^Tc-Sestamibi also enables interrogation of mitochondrial potential similar to that of TMRE and Rhodamine 123 recently has been used to evaluate anthracycline-induced cardiotoxicity [24] in rodents. However, myocardium retention may need to be corrected using either pharmacokinetic modeling or normalized to another FDA-approved tracer to determine net signal in myocytes for diagnosis of cardiotoxicity. Compared with SPECT, PET tracers provide high sensitivity and enable quantification, thus allowing 3-dimensional pharmacokinetic analysis. Therefore, taking advantage of principles of lipophilic cations, ^18^F-Mitophos, a triphenylphosphonium analogue, has been recently evaluated for its potential to image DOX-induced cardiotoxicity in rat models [25••]. The agent may be susceptible to concerns of perfusion effects similar to 99mTc-Sestamibi and also indicates significant metabolism, thus indicating reduction of parental tracer within minute post administration in vivo. Furthermore, ^18^F-DHMT, a dihydroethidium derivative [26••, 27], has been also evaluated to monitor anthracycline-induced ROS cardiotoxicity in vivo [28••]. Compared with significant effects observed in LVEF at 6-week post DOX-treatment, ^18^F-DHMT enables detection of superoxide production at 4-week post treatment [28••]. Following further validations, the PET agent may allow opportunities for quantifying therapeutic efficacy of cardioprotectants, such as Dexrazoxane, an FDA-approved drug for late stage treatment of breast cancer patients [29].

While ^99m^Tc-incorporated radiotracers have been the workhorse in nuclear medicine for decades due to commercial availability of ^99^Mo/^99m^Tc generators in nuclear pharmacies, however, disruptions in the supply chain during the ^99^Mo/^99m^Tc crisis of 2008–2010 demonstrated the vulnerability of the world supply of ^99^Mo. To address these shortcomings, over the last decade, germanium/gallium (Ge/Ga) generators have become available and are capable of producing high quality ^68^Ga (t_½_   =  68 min), an isotope with excellent emission properties for clinical PET imaging [30]. The parent isotope, ^68^Ge (*t*_½_   =  271 days), is produced in high-energy proton accelerators from a ^69^Ga(p,2n)^68^Ge reaction and is bonded to alumina for eventual elution on-site [31], thus providing a practical generator-based distribution model for on-site formulation of PET radiopharmaceuticals. With co-development of high-quality ^68^Ga-based tracers, PET imaging could be unlinked from proximity to cyclotrons, thereby expanding access to the technology. In next section, we discuss development of another molecular imaging agent, described in literature as ^68^Ga-Galmydar, to track anthracycline induced effects in cellulo and in vivo.

Importantly, ^68^Ga-Galmydar is also recognized by ATP-binding-cassette (ABC) family of transporters, such as ABCB1 (also known as P-glycoprotein, 170 kD protein located on plasma membrane of tumor cells) and ABCG2 (also known as breast cancer resistance protein, BCRP, 72 kD protein) as their transport substrate [32]. Overall, the net retention of ^68^Ga-Galmydar in heart tissue is determined by the opposing action of two biochemical processes [33]. Deploying delocalization of charge on its molecular surface, this tracer permeates passively into living cells and concentrating within the mitochondrial inner matrix in response to the driving forces of electronegative plasma membrane and mitochondrial transmembrane potentials [34]. However, this uptake is opposed by action of ABC membrane transporters, such as *ABCB1*, MRP1 (*ABCC1*), and ABCG2, which excrete the radiotracer into extracellular space of tumor cells [35–39]. Importantly, cardiomyocytes, although rich in mitochondria, they lack expression of efflux transporter proteins, thus sequestering this radiotracer for prolonged periods to enable imaging, while hepatocytes, which express ABCB1 and ABCG2 along their cannalicular surface, rapidly excrete it into the bile and intestines. In principle, these biochemical traits would be expected to facilitate hepatocellular clearance of the tracer, thereby minimizing the impact of γ-emissions arising from the liver that could potentially co-register into the inferior wall of the myocardium during imaging.

**Fig. 1:**
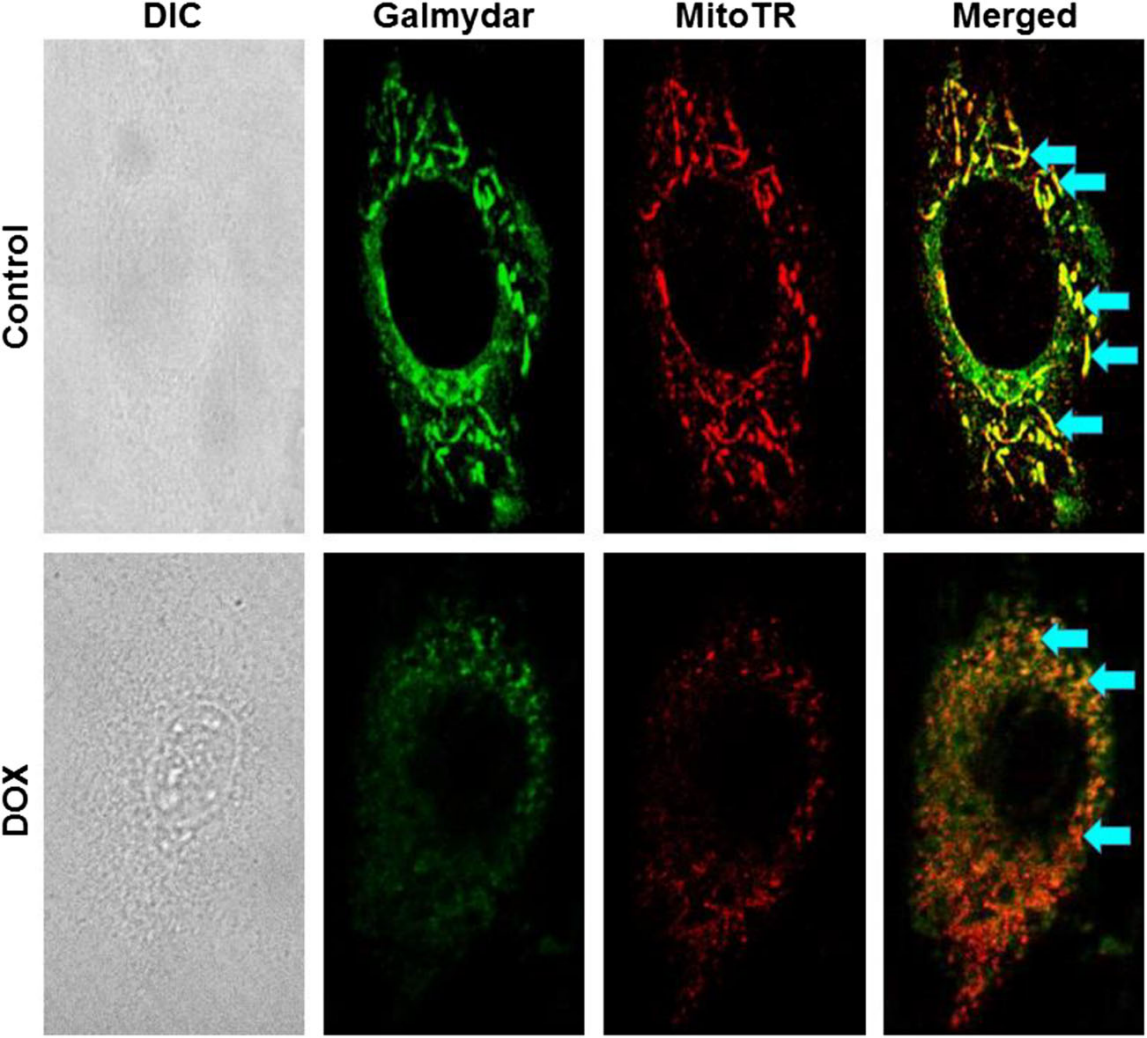
Intracellular localization of Galmydar in mitochondria of rat cardiomyoblasts correlation via with Mito-Tracker Red: Images were acquired using a 60 X objective (all panels represent same magnification) in live H9c2(2–1) cells following 30-min treatment with Galmydar (20 μM) and MitoTracker Red CM-H2XRos (25 nM). Control (top panel); DOX treated (Lower Panel). *Arrows* depict localization within mitochondria. (Reproduced from: Sivapackiam J, et al. PLoS One May 2019 23;14(5):e0215579. doi: 10.1371/journal.pone.0215579. eCollection 2019; Creative Commons user license https://creativecommons.org/licenses/by/4.0/) [45••]

**Fig. 2:**
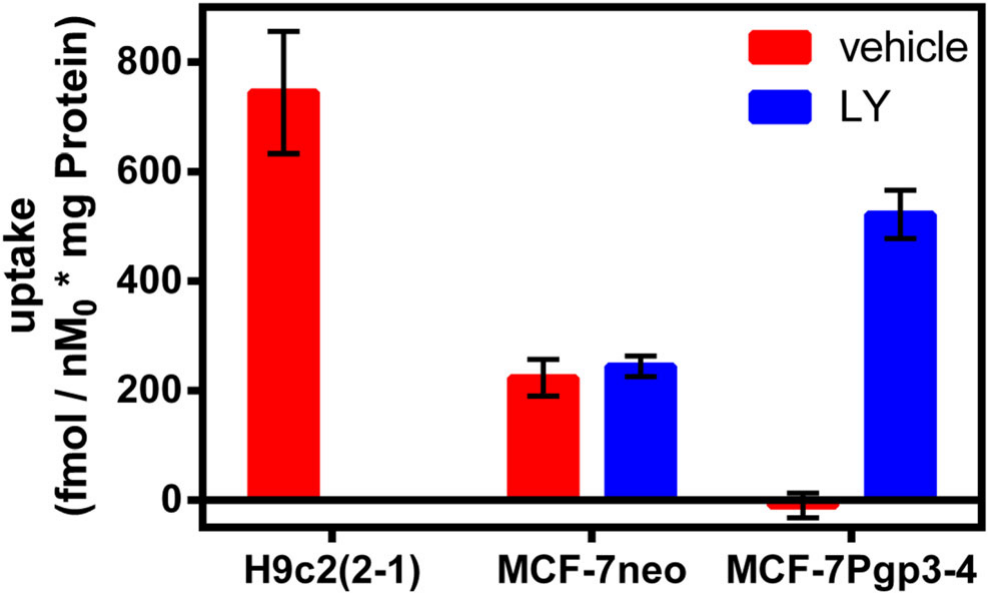
Characterization of ^68^Ga-Galmydar, in cardiomyoblasts H9c2(2–1) and human breast carcinoma (MCF-7neo (WT) including stably transfected counterparts MCF-7Pgp3–4) cells: shown is net uptake at 90 min (fmol × (nM_0_)^−1^ × (mg protein)^−1^) using a control buffer either in the absence or presence of LY335979, a highly specific and sensitive antagonist of ABCB1(1 μM). Each bar represents the mean of 4 determinations; lines above and below the bar denote ±SD. (Reproduced from: Sharma V, et al. PLoS One 2014;9(10):e109361); Creative Commons user license https://creativecommons.org/licenses/by/4.0/) [39]

**Fig. 3:**
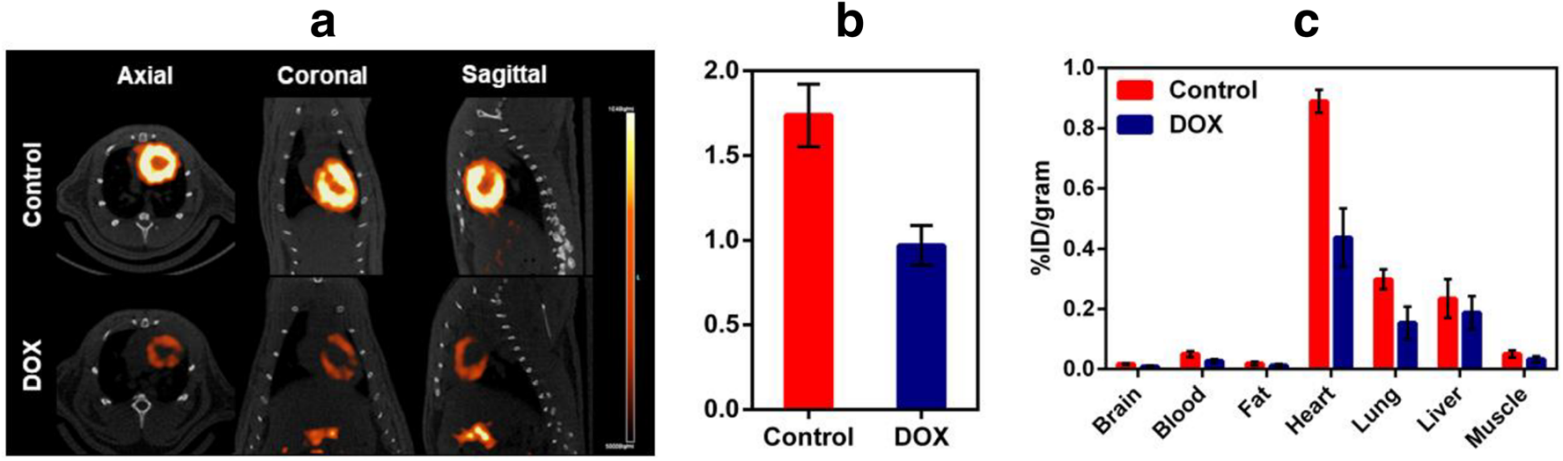
**a** Micro-PET/CT imaging. Sprague-Dawley (SD) rats were injected intravenously with ^68^Ga-Galmydar, and static PET images were acquired for 10-min, 60-min post tail-vein injection. Top panel: Control rat; lower panel: DOX (15 mg/kg, 5 days prior to imaging)-treated rat. Similar results were obtained in 3 independent experiments. **b** SUV analysis of ^68^Ga-Galmydar uptake in hearts of SD rats (mean ± SD, *n* = 3). **c** Post-Imaging biodistribution data (%ID/g) for ^68^Ga-Galmydar in rats treated either with DOX (15 mg/kg; 5 days prior to imaging) or vehicle as a control (mean ± SD, *n* = 3). (Reproduced from: Sivapackiam J, et al. PLoS One May 2019 23;14(5):e0215579. doi: 10.1371/journal.pone.0215579. eCollection 2019; Creative Commons user license https://creativecommons.org/licenses/by/4.0/) [45••]

Galmydar is also a mildly fluorescent molecular imaging probe and localizes within the mitochondria of rat cardiomyoblasts (H9c2) similar to mitotracker Red [40–42] (Fig. 1**)**. While tracer shows stable accumulation in rat cardiomyoblasts, its uptake profiles in MCF-7 cells are inversely proportional to expression of ABCB1 on the plasma membrane (Fig. 2) [39]. Similar uptake profiles are observed in HEK 293 ABCG2 transfected cells [32, 43, 44]. The agent shows dose and time-dependent pharmacological response to anthracycline in rat cardiomyoblasts [45••], using live cell imaging, thereby consistent with postulated mechanism of anthracycline-induced depolarization of mitochondrial redox potentials, and ROS production [46]. To further assess potential of ^68^Ga-Galmydar to serve as a molecular imaging probe for assessment of DOX-induced cardiotoxicity in vivo, ^68^Ga-Galmydar has been injected via tail-vein into rats pretreated either with intravenous administration of DOX (15 mg/kg) or vehicle (5% ethanol in saline). Following treatments, micro-PET static scans (10-min acquisition; 60-min post tail-vein administration of ^68^Ga-Galmydar; Fig. 3a) demonstrated a 1.91-fold lower retention in hearts of DOX-treated (Standard Uptake Value; SUV: 0.92, *n* = 3) rats compared with their vehicle-treated counterparts (SUV: 1.76, *n* = 3; Fig. 3b) [45••]. For correlation of PET data, post-imaging quantitative biodistribution studies demonstrate heart retention values of 2.02-folds lower for DOX treated (%ID/g; DOX: 0.44 ± 0.1, *n* = 3) rats compared to their vehicle-treated counterparts (vehicle control: 0.89 ± 0.03, *n* = 3, *p* = 0.04; Fig. 3c**)**, thus supporting micro-PET imaging data in vivo [45*••*]. Of note, using live-cell imaging, Galmydar also indicate a gradual depression in cellular uptake and retention of Galmydar (up to 8.2-fold difference compared to their untreated cells after 5 h), thus indicating the sensitivity of the probe to map changes at the level of the mitochondria resulting from DOX treatment, which in turn likely result from depolarization of the mitochondrial potential [45••]. These findings are consistent with literature precedents, wherein DOX treatment has been shown to alter mitochondrial redox potentials, thus depolarizing mitochondria, and elevating matric Ca2+ and ROS production in 30 min [46]. Finally, high-resolution single-cell imaging also shows localization of Galmydar in mitochondria of DOX-treated cells (Fig. 1, lower panel) similar to their untreated controls (top panel), however, substantial decreased retention thus correlating with the lower PET signal in heart of DOX-treated rats (Fig. 3**)**. Following further validations in higher vertebrates, ^68^Ga-Galmydar imaging could enable monitoring of impaired mitochondrial function in myocytes following anthracycline treatment in vivo.

## Conclusions

While a significant loss in contractile function of the myocardium may serve as a warning for irreversible tissue damage, current imaging techniques may not have the desired sensitivity and molecular specificity to guide interventions at early stages of cardiotoxicity. Among various imaging modalities, nuclear imaging-based strategies can potentially be translated faster into clinic due to the need for administration of doses at very low concentrations. Both mitochondrial potential- and ROS-targeted tracers may allow noninvasive imaging of anthracycline-induced cardiotoxicity in vivo. Because frontiers of molecular imaging in twenty-first century are pushing the edge of the envelop to detection at earliest stages, it may be argued biochemically that changes in the mitochondrial potentials represent an upstream event, before triggering the production of the ROS and caspase activity; thus, it is conceivable that tracers capable of reporting changes in the mitochondrial potential in vivo might offer interrogation of cardiotoxicity at earliest stages as evident from imaging of ^18^F-Mitophos and ^68^Ga-Galmydar in rodent models. It remains to be determined, whether these initial observations would replicate in higher vertebrates and translate into humans. We envision that both categories of radiotracers could be beneficial for monitoring cardiotoxicity in the field of cardio-oncology and may provide opportunities for interrogating therapeutic efficacy of cardio-protectants, while offering opportunities for stratification of cancer patients for modification of therapeutic protocols.
